# iNOS promotes hypothalamic insulin resistance associated with deregulation of energy balance and obesity in rodents

**DOI:** 10.1038/s41598-017-08920-z

**Published:** 2017-08-23

**Authors:** Carlos Kiyoshi Katashima, Vagner Ramon Rodrigues Silva, Luciene Lenhare, Rodrigo Miguel Marin, José Barreto Campello Carvalheira

**Affiliations:** Department of Internal Medicine, University of Campinas, UNICAMP, Campinas, SP Brazil

## Abstract

Inducible nitric oxide (iNOS)-mediated S-nitrosation of the metabolic signaling pathway has emerged as a post-translational modification that triggers insulin resistance in obesity and aging. However, the effects of S-nitrosation in controlling energy homeostasis are unknown. Thus, in the present study we aimed to evaluate the effects of S-nitrosation in insulin signaling pathway in the hypothalamus of rodents. Herein, we demonstrated that the intracerebroventricular infusion of the nitric oxide (NO) donor S-nitrosoglutathione (GSNO) promoted hypothalamic insulin signaling resistance and replicated the food intake pattern of obese individuals. Indeed, obesity induced S-nitrosation of hypothalamic IR and Akt, whereas inhibition of iNOS or S-nitrosation of insulin signaling pathway protected against hypothalamic insulin resistance and normalized energy homeostasis. Overall, these findings indicated that S-nitrosation of insulin signaling pathway is required to sustain hypothalamic insulin resistance in obesity.

## Introduction

Originally appreciated as a reservoir of excess calories, research over the last 20 years has demystified adipose tissue as an important endocrine organ. Currently, adipose tissue is considered not only a tissue specialized in energy storage, but also a dynamic sensor of body energy balance that secretes adipokines to integrate metabolic homeostasis^1–3^. Leptin and insulin, whose levels are proportional to adiposity, signal distinct neuronal populations in the arcuate nucleus to modulate, in a coordinated manner, the expression of orexigenic and anorexigenic neuropeptides^2, 4, 5^. As a result, the information gathered from adipose tissue is transmitted to a complex neural network that manages food intake, energy expenditure and metabolism^6, 7^. Despite the fact that energy homeostasis is tightly controlled, and relies on a fine-tuned neural circuit, the mechanism by which overnutrition disrupts this system remains unclear.

Obesity physiopathology is now widely recognized as being strictly associated with low-grade inflammation^8–10^. Mechanistically, inflammatory immune cells infiltrate the adipose tissue of obese individuals and overcome the protective effects of resident leukocytes to promote and sustain inflammation and insulin resistance^11, 12^. Recently, a similar inflammatory milieu was found to concomitantly occur in the hypothalamus of obese individuals^13, 14^. For instance, obese rats were found to present increased levels of hypothalamic JNK, which represses insulin signaling in the central nervous system^15, 16^. Accordingly, specific inhibition of pro-inflammatory proteins such as TNF, TLR4 and IKKβ in the hypothalamic tissue of obese animals restores leptin sensitivity^17–19^. The opposite was observed when IL-10 is inhibited^20, 21^. In aggregate, these studies shed light on hypothalamic inflammation as an important modulator of energy homeostasis, which sustains leptin and insulin resistance and promotes obesity.

Nitrosative stress is a hallmark of inflammation and is causally linked with inducible nitric oxide synthase (iNOS)^22, 23^. Not surprisingly, iNOS induced nitrosative stress, such as S-nitrosation and nitration, has emerged as a potent modulator of the insulin-signaling pathway in obesity^24–28^. Accordingly, S-nitrosation of IR, IRS-1 and Akt have been linked to insulin resistance in obesity and aging, whereas exercise has been shown to reverse S-nitrosation and sustain insulin sensitivity in the muscle of obese animals^24, 29^. While these studies revealed a role of S-nitrosation in liver and muscle insulin resistance, the role of this post-translational modification in other metabolic tissues is still unknown.

Morley and Flood^30^ showed that subcutaneous injection of L-N-nitro arginine, an inhibitor of endogenous nitric oxide (NO) synthesis, leads to a dose-dependent decrease in food intake in mice. Conversely, food intake was increased with injections of L-arginine, indicating that NO action in the central nervous system may regulate appetite. Accordingly, inhibition of nitric oxide synthase (NOS) impairs the orexigenic effects of NPY and ghrelin^31, 32^. Furthermore, it has been previously reported that iNOS levels are increased in the hypothalamus in an adiposity dependent fashion^33^. However, the role of nitrosative stress in insulin hypothalamic-signaling pathway is unclear. We report herein that the ICV administration of the NO donor, S-nitrosoglutathione (GSNO), not only induces S-nitrosation and inhibits hypothalamic insulin signaling pathway, but also increases food intake. Moreover, we show that obesity promotes S-nitrosation of insulin signaling pathways while inhibition of iNOS restores hypothalamic insulin signaling. Altogether, these data highlight an unprecedented role for S-nitrosation in the regulation of body weight.

## Results

### Effects of the NO donor, S-nitrosoglutathione (GSNO) on energy homeostasis and insulin signaling in mice

**Figure 1:**
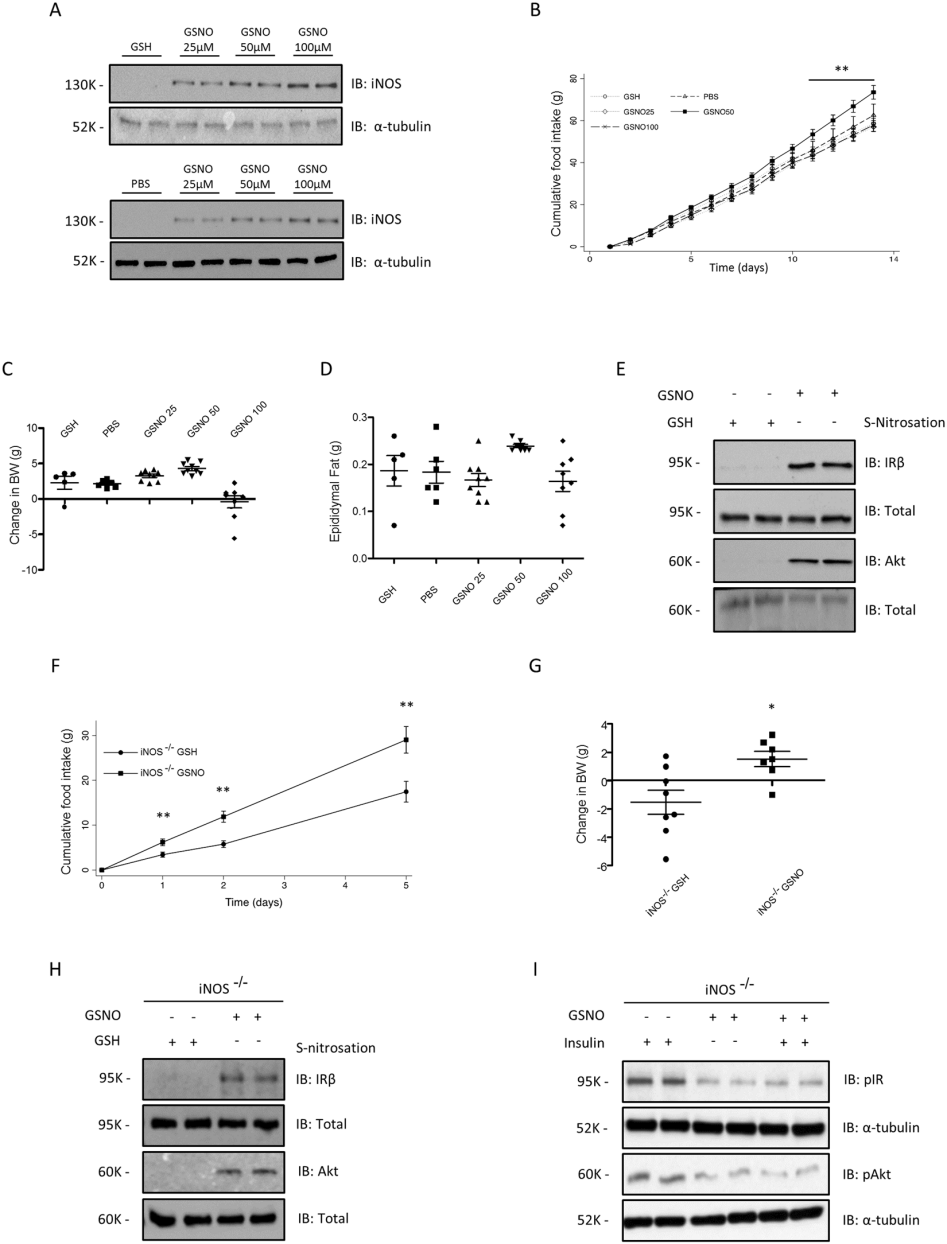
ICV GSNO modulates energy homeostasis. Immunoblot analysis for total iNOS in hypothalamus animals treated with GSNO (**A**) Cumulative food intake (**B**), body weight change (**C**) and epididymal fat weight (**D**) of GSH or GSNO treated mice. Biotin switch method analysis for IRβ and Akt S-nitrosation in mice treated with GSNO (**E**). Cumulative food intake (**F**) and body weight change (**G**) in GSH, PBS  or GSNO treated iNOS knockout mice. Biotin switch method analysis for IRβ and Akt S-nitrosation in iNOS knockout mice treated with GSNO (**H**) Western Blotting analysis for insulin-induced phosphorylation of IRβ and Akt in iNOS knockout mice treated with GSH or GSNO (**I**). Data are presented as mean ± SEM, *p < 0.05 vs. GSH -injected rats; **p < 0.01 vs. other groups (n = 10–15 animals per group).

To investigate the role of S-nitrosation in energy homeostasis, we implanted mini-osmotic pumps into the third ventricle of rats to continuously administer GSNO for one week. GSNO is a low-weight primary S-nitrosothiol, which is capable of transferring the nitrosonium cation (NO^+^) to cysteine residues of proteins via transnitrosation reactions, modulating their activity^34^. In order to evaluate the stability of GSNO in the hypothalamus we measured the amount of GSNO decomposed after 20 and 60 min of incubation with hypothalamic tissue homogenate. Our results showed that the amount of GSNO decomposed in this medium was statistically equal to the amount decomposed in PBS solution after the same time period. This result shows that GSNO decomposition is not accelerated by hypothalamic enzymes (Figure S1A). Continuous ICV infusion of GSNO promoted increase in iNOS levels in a dose dependent manner (Fig. 1A), in contrast we observed a increase in food intake after 14 days and a trend in weight gain in C57BL6 mice, which received 50 μM of GSNO, but we did not detect an effect of GSNO 25 μM or 100 μM (Fig. 1B and C). We did not detect changes in epididimal fat weight (Fig. 1D). In parallel we observed that GSNO 50 μM induced S-nitrosation of the crucial proteins, IR and Akt, for insulin signaling IR and Akt (Fig. 1E).

Consistent with an S-nitrosation mediated effect, continuous ICV infusion of GSNO 50 μM in iNOS knockout mice resulted in an overt increase of food intake and weight gain (Fig. 1F and G), which was associated with an increase in insulin and IL-6 levels (Table S2). We next determined if the GSNO 50 μM induced modulation of weight gain was associated with the disruption of hypothalamic insulin sensitivity. GSNO 50 μM induced S-nitrosation of IR and Akt in mice (Fig. 1H) and inhibited insulin signaling, as measured by the phosphorylation levels of these proteins (Fig. 1I). Therefore, GSNO 50 μM reverted the tolerogenic effect of decreased iNOS expression.

### ICV GSNO 50 μM treatment leads to hypothalamic insulin resistance in rats

**Figure 2:**
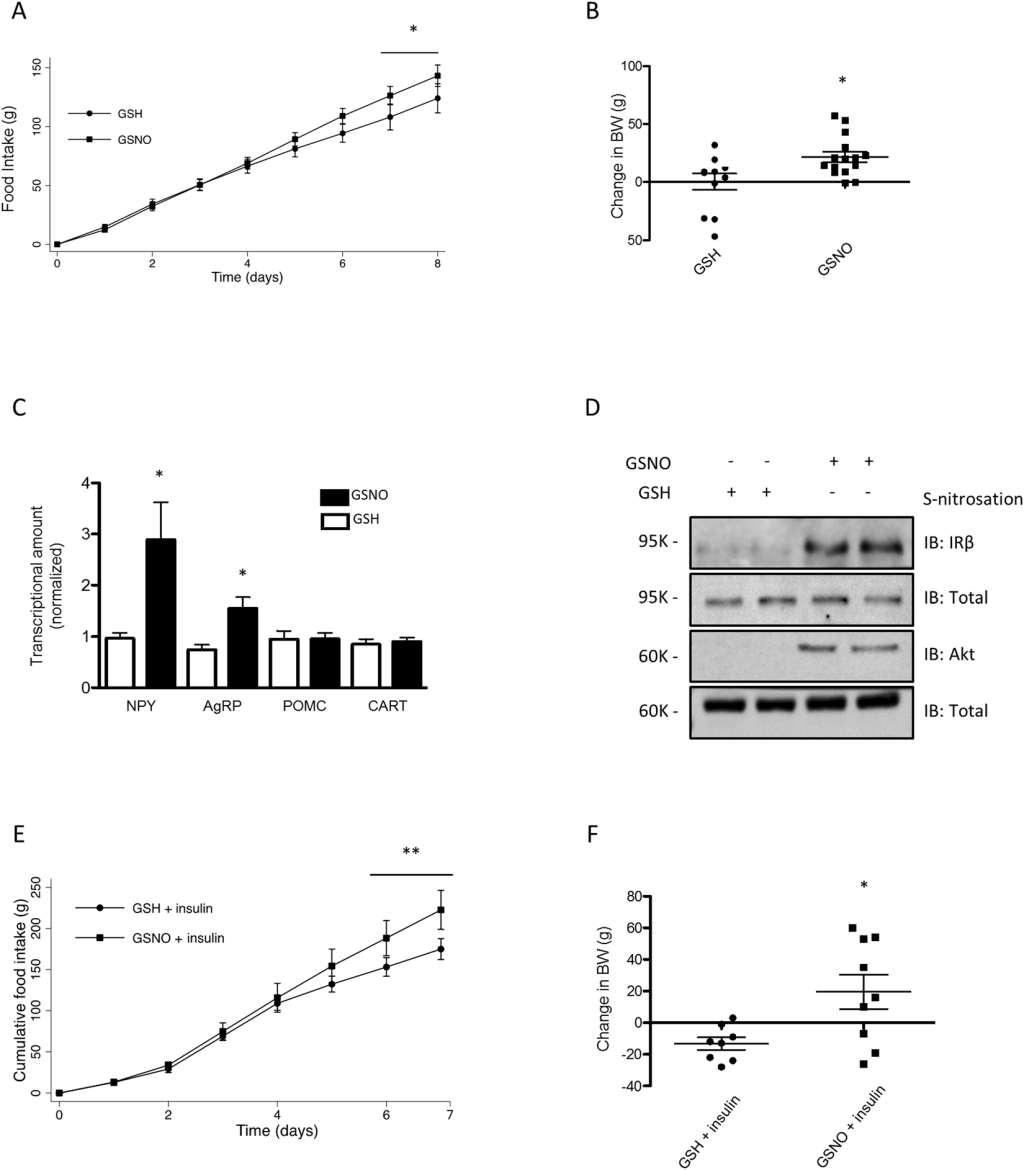
GSNO induces insulin resistance in hypothalamus. Cumulative food intake (**A**) and body weight change (**B**) of GSH or GSNO treated rats. Real-time analysis of NPY, AgRP, POMC and CART, expression generated from GSH or GSNO treated rats (**C**). Biotin switch method analysis for IRβ and Akt S-nitrosation in rats treated with GSNO (**D**). Cumulative food intake (**E**) and body weight change (**F**) in insulin or insulin plus GSNO treated rats. Data are presented as mean ± SEM, *p < 0.05 vs. GSH -injected rats; **p < 0.01 vs. GSNO treated group (n = 10–15 animals per group).

Similarly, chronic administration of GSNO 50 μM resulted in an increase of food intake and weight gain in rats (Fig. 2A and B). In parallel we detected an increase in insulin and leptin as well as reduced adiponectin levels in animals treated with GSNO 50 μM (Table S2). However, we did not detect changes in body mass and epididymal fat pad weight as well as in pro-inflammatory cytokines (Table S2). Interestingly, we failed to detect changes in activity levels of GSNO 50 μM - treated rats and observed a mild increase in energy expenditure as indicated by VO_2_, and VCO_2_ measurements during the light cycle (Table S2) suggesting that GSNO 50 μM effects are mainly related to the control of food intake. Accordingly, GSNO 50 μM induced a significant increase of orexigenic neuropeptides, *NPY* and *AgRP*, while the levels of the anorexigenic neuropeptides, *POMC* and *CART*, did not change after GSNO treatment (Fig. 2C). While GSNO 50 μM induced S-nitrosation of IR and Akt (Fig. 2D).

In accordance with a GSNO-induced hypothalamic insulin resistance, GSNO 50 μM suppressed insulin-induced decrease in food intake and body weight in rats (Fig. 2E and F). These findings indicated that pharmacological induction of S-nitrosation in insulin signaling pathways is associated with the repression effect this hormone has on food intake and raises the question of whether S-nitrosation is an important event in the pathophysiology of obesity.

### Effects of diet induced obesity (DIO) and exercise on IR and Akt S-nitrosation in rat hypothalamus

**Figure 3:**
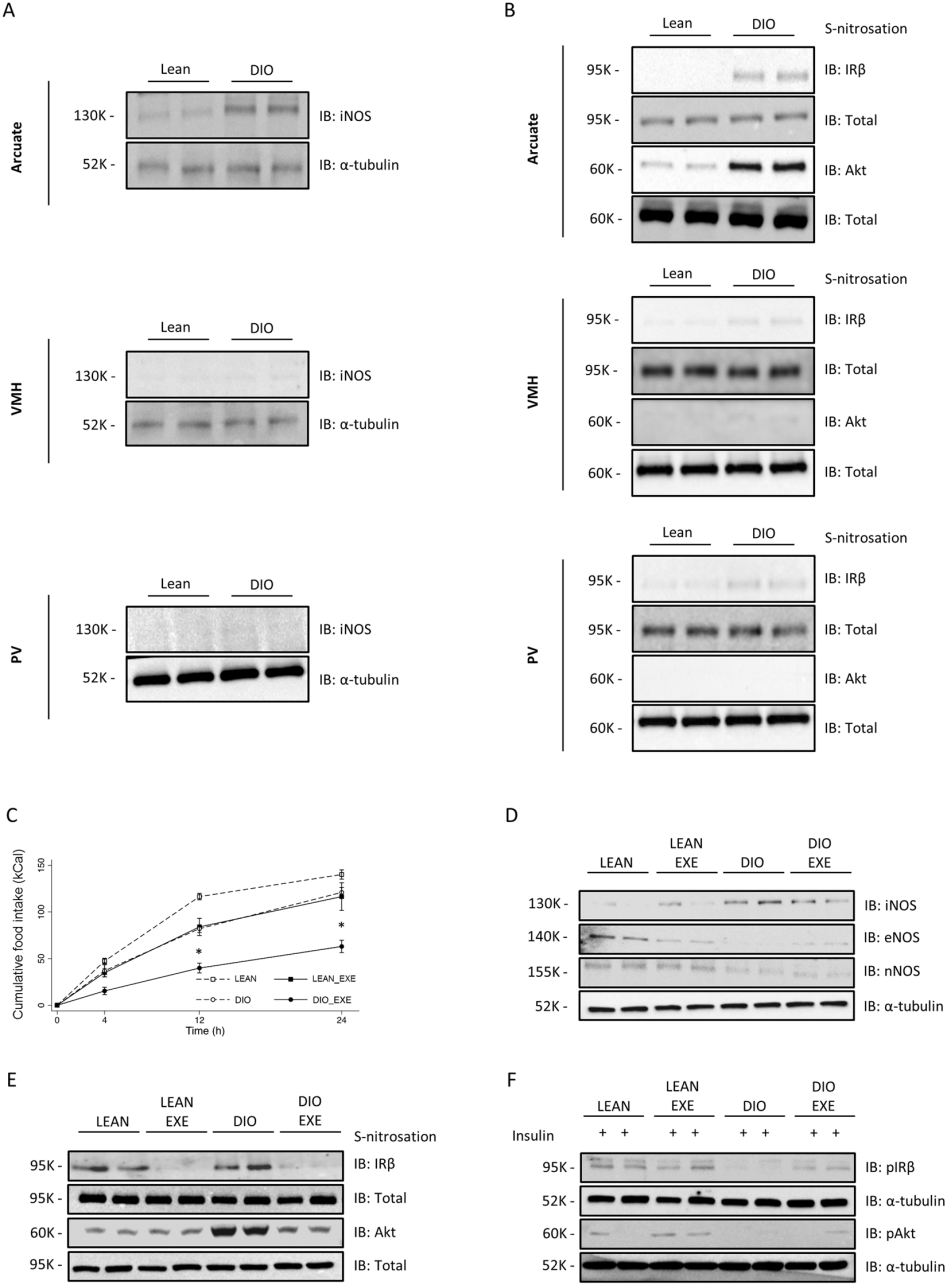
Exercise reverses insulin S-nitrosation in hypothalamus and normalizes energy homeostasis. Immunoblotting analysis of iNOS expression generated from lean and DIO rats in distinct hypothalamic nuclei (**A**) The biotin switch method was used to determine IRβ and Akt S-nitrosation in arcuate, ventromedial (VMH) and paraventricular (PV) nuclei of lean and DIO rats (**B**) (n = 10 animals per group). Effects of exercise on food intake (**C**). Immunoblotting analysis of iNOS, eNOS and nNOS expression generated from lean and diet induced obesity (DIO) rats submitted to physical exercise (**D**). The biotin switch method was used to determine IRβ and Akt S-nitrosation in hypothalamus of exercised lean and DIO rats (**E**). Western Blotting method was used to determine insulin-induced phosphorylation of IRβ and Akt (**F**). Data are presented as mean ± SEM, *p < 0.05 vs. lean groups (n = 8–10 animals per group).

In order to investigate the levels of iNOS and the S-nitrosation status of the insulin pathway, we systematically dissected the hypothalamus and its nuclei from lean and obese animals. DIO rats showed a robust increase in arcuate nucleus iNOS levels, while we could not detect iNOS in VMH and a faint iNOS expression was detected in PV (Fig. 3A). Diet induced obesity (DIO) rats showed a robust increase in arcuate nucleus iNOS levels, while we could not detect iNOS in VMH and a faint iNOS expression was detected in PV (Fig. 3A). In parallel, we detected increased S-nitrosation levels in IR and Akt in the arcuate nucleus, whereas there was a small amount of IR nitrosation and no changes in S-nitrosation of Akt in VMH and PV (Fig. 3B). These data suggest that obesity-induced S-nitrosation in the insulin signaling pathway is important in the orchestration of energy homeostasis.

The above results raised the question of whether reducing hypothalamic iNOS levels in obese animals restores food intake to lean animal settings. This was firstly addressed by taking advantage of the hypothalamic anti-inflammatory effects of physical activity^35^. A single bout of exercise reversed the increased food intake observed in DIO rats (Fig. 3C) and decreased body weight without changing their epididymal fat content (Figure S2A-B). In accordance with these results, physical activity robustly decreased hypothalamic levels of iNOS and S-nitrosation of IR and Akt (Fig. 3D and E). Importantly, DIO was associated with a decrease in eNOS and nNOS and exercise did not modulate the level of these enzymes (Fig. 3D). Simultaneously, we observed that exercising DIO rats restored insulin-induced phosphorylation of IR and Akt to lean control levels (Fig. 3F). These findings suggest that hypothalamic iNOS levels and S-nitrosation of insulin signaling pathway are physiologically regulated and modulate hypothalamic sensing of adiposity.

### Disruption of hypothalamic iNOS prevents obesity in DIO rodents

**Figure 4:**
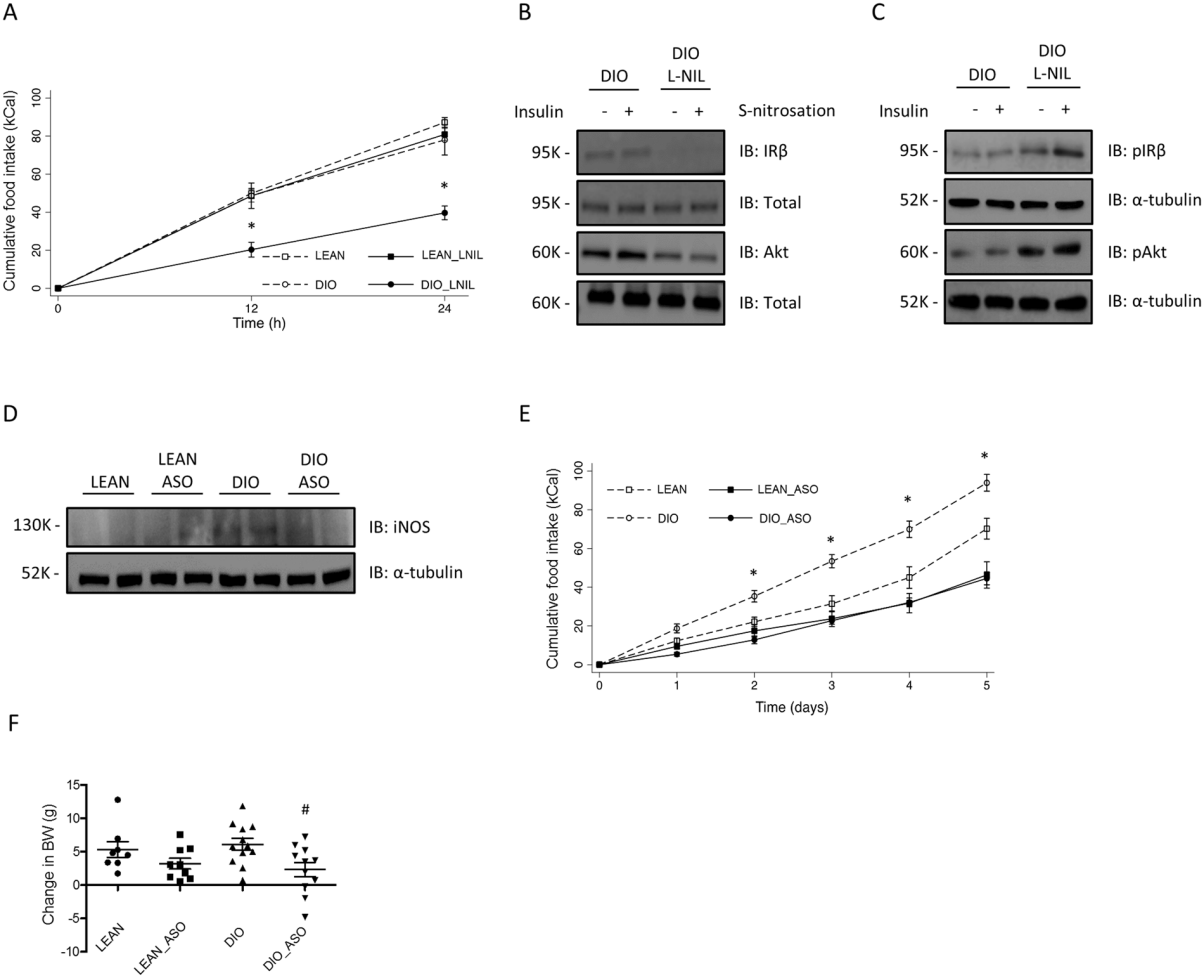
Pharmacological and genetic inhibition of iNOS reverses S-nitrosation in hypothalamus and normalizes energy homeostasis. Effects of L-NIL on food intake in hypothalamus of L-NIL treated lean and DIO rats (**A**). The biotin switch method was used to determine IRβ and Akt S-nitrosation in hypothalamus DIO and DIO_L-NIL (**B**). Western Blotting method was used to determine insulin-induced phosphorylation of IRβ and Akt in rats treated with vehicle or L-NIL in lean and DIO rats treated with L-NIL (**C**). Effects of iNOS antisense oligonucleotide (ASO) (**D**) on food intake (**E**), body weight change (**F**) in lean and DIO mice. Data are presented as mean ± SEM, *p < 0.05 vs. vehicle-injected rats; ^#^ *p* < 0.05 vs. respective lean group (n = 6–8 animals per group).

To further test this hypothesis, we pharmacologically inhibited iNOS activity in the hypothalamus of DIO rats. Congruent with this idea, ICV administration of L-NIL drastically reduced food intake in obese rats without affecting food intake in lean animals (Fig. 4A). Consistent with the inhibition of iNOS activity, L-NIL administration reduced obesity-mediated S-nitrosation of IR and Akt (Fig. 4B). In parallel, we observed restoration of insulin-induced phosphorylation of IR and Akt in DIO animals treated with L-NIL (Fig. 4C). In an effort to elucidate the effects of genetic inhibition of iNOS in the control of food intake, we inhibited the expression of this enzyme in the hypothalamus by knocking down iNOS with a specific oligonucleotide antisense (ASO). ASO treatment promoted a reduction in iNOS levels in DIO-fed mice, which was accompanied by a significant decrease in food intake and change in body weight (Fig. 4D–F).

## Discussion

This study provided evidence that iNOS induced S-nitrosation of intracellular metabolic pathways is a ubiquitous process promoted by the low-grade inflammatory environment of obesity. As previously demonstrated, S-nitrosation of the insulin signaling pathway promotes insulin resistance in hepatic and muscular tissue^28, 29^. We extended these findings by demonstrating that S-nitrosation orchestrates food intake by sustaining hypothalamic insulin resistance and promoting obesity.

There are a number of molecular events that are causally linked to impairment of insulin-signaling pathway in the hypothalamus of obese individuals^15, 36^. S-nitrosation is a post translational modification induced by NO, that similarly to phosphorylation, occurs in a broad number of proteins and is controlled by denitrosylating enzymes, which act in similar fashion to the protein phosphatase that controls phosphorylation^37, 38^. Here we demonstrated that increased hypothalamic S-nitrosation of the insulin signaling pathway may be one of these molecular mechanisms. S-nitrosation of IR and Akt proteins is believed to be an important mechanism in repressing insulin-signaling activity^24, 27, 29^. Moreover, the increased hypothalamic S-nitrosation of IR and Akt was linked to decreased phosphorylation of these enzymes, which is consistent with the impairment of their enzymatic activity, as previous found^27^. Accordingly, the reversion of S-nitrosation by physiological, pharmacological and genetic approaches resulted in reversion of insulin resistance. Taken together, these findings suggest that obesity mediates insulin resistance, at least in part, by inducing S-nitrosation of IR and Akt.

The association between NO and energy homeostasis has long been known. Pharmacological modulation of hypothalamic NOS activity promotes robust changes in food intake and energy homeostasis^30^. Moreover, inhibition of NOS resulted in weight loss in *ob/ob* mice^39^ while leptin and NPY treatment increased hypothalamic NO levels suggesting it plays the role of a neurotransmitter^32^. We showed that GSNO 50 μM -induced S-nitrosation of insulin signaling pathways promotes hypothalamic resistance to these hormones and increases food intake, leading to an anabolic state. Interestingly, obese rodents have increased levels of hypothalamic iNOS and S-nitrosated insulin signaling pathways. Accordingly, inhibition of iNOS expression or activity by physiological, pharmacological or genetic approaches, restored food intake to lean settings. Therefore, our findings revealed a novel role for NOS in the control of food intake, showing that iNOS-induced S-nitrosation of insulin signaling pathway induces chronic changes in energy homeostasis, which promotes obesity.

Obesity induced neuroinflammation was recently integrated into the physiopathology of obesity^13, 40^. In spite of the increased expression of iNOS in a variety of conditions associated with acute or chronic inflammation, the pathophysiological role of iNOS is complex and sometimes dichotomic^22, 23^. Interestingly, S-nitrosation has been linked to the anti-inflammatory effects of NO. Specifically, S-nitrosation decreases the activity of pro-inflammatory molecules such as JNK^41^, IKKβ^42^ and NFkB^43^, which are also associated with obesity-mediated neuroinflammation and neurodegeneration^17, 44^. Thus, collectively, our findings suggest that S-nitrosation of IR and Akt supersedes its potential anti-inflammatory effects and therefore contributes to obesity physiopathology. Importantly, exercise-mediated neurological control of weight loss has been associated with attenuation of hypothalamic inflammation and protection of POMC neurons from apoptosis, resulting in increased insulin and leptin sensitivity^45–48^. Consistent with these results we observed that exercise decreased S-nitrosation of IR and Akt in hypothalamus and reduction in food intake of DIO mice.

Besides the effects of hypothalamus in energy balance, this organ has also emerged as a fine-tuning regulator of glucose and lipid peripheral metabolism^49–52^. Different approaches to generate isolated neuronal insulin resistance invariably resulted in increased adiposity^49, 53, 54^. In agreement with these studies, icv administration of GSNO 50 μM induced insulin resistance resulted in increased body weight, leptin levels and peripheral insulin resistance. Interestingly, icv GSNO 50 μM infusion decreased triglycerides (TG) levels in rats in spite of peripheral insulin resistance whereas increased TG levels were observed in iNOS knockout mice. Given that hepatic VLDL-TG secretion is susceptible to the CNS administration of distinct molecules such as fatty acids^52^, NPY^55^, glycine^56^ and glucose^50^ it is possible that GSNO 50 μM infusion direct modulates lipid metabolism in a rodent dependent fashion.

The results presented herein do not exclude the possibility that besides S-nitrosation, nitration could also be involved in iNOS induced hypothalamic insulin resistance. In this context, Charbonneau and Marette showed that the covalent link of peroxynitrite (ONOO^-^) to IR, IRS-1, IRS-2 and Akt is also involved with insulin resistance after lipid infusion^25^. Along this line, JAK2 nitration has been associated with growth hormone resistance^57, 58^. Therefore, it is reasonable to speculate that these two post-translational phenomena cooperate to disrupt insulin signaling. However, the effects of nitration in the control of energy balance are unknown and deserve further investigation.

Nitric oxide has been associated with a bimodal effect in peripheral tissues. Specifically, in the liver the inhibition of NO synthase activity leads to insulin resistance^59^, whereas, the activation of iNOS leads to insulin resistance^24^. Given that the observed effects on the control of food intake in our study was limited to GSNO dose of 50 μM, our results do not refute the possibility that GSNO could have distinct effects on hypothalamic insulin sensitivity in a similar fashion to that observed in peripheral tissues.

In summary, we have demonstrated a central role for iNOS and S-nitrosation in the control of energy homeostasis. In obese animals, iNOS-induced S-nitrosation of insulin signaling pathway robustly repressed the signaling of this hormone and promoted increased food intake. In aggregate, these findings suggest that inhibiting S-nitrosation of insulin signaling pathway may be a valuable ally in treating obesity and its related diseases. However, little is known about the mechanisms that counter-regulate S-nitrosated insulin signaling proteins. Thus, in the future, it will be crucial to better understand the mechanisms whereby hypothalamic S-nitrosation is modulated.

## Methods

### Antibodies and chemicals

Nitrocellulose paper (Hybond ECL, 0.45 mm) was supplied by Amersham Pharmacia Biotech United Kingdom Ltd. (Buckinghamshire, United Kingdom). Ketamine was from Parke-Davis (São Paulo, SP, Brazil), diazepam and thiopental were from Cristália (Itapira, SP, Brazil). Anti-phospho-IRβ (rabbit polyclonal, SC-25103), anti-IRβ (rabbit polyclonal, SC-711) and NOS2 (mouse polyclonal, SC-7271) antibodies were from Santa Cruz Biotechnology, Inc. Anti-phospho-Akt (rabbit polyclonal, #9271), anti-alpha tubulin (rabbit polyclonal, #2144), and anti-Akt (rabbit polyclonal, #9272) were from Cell Signaling Technology (Beverly, MA, USA). Insulin came from Eli Lilly and Company (Indianapolis, IN, USA). Routine reagents were purchased from Sigma Chemical Co. (St. Louis, MO, USA).

### Animals and diets

Male 4-week-old Wistar rats, Swiss, C57BL/6 and iNOS-null (*iNOS* ^*−/−*^) mice (C57BL/6-*Nos*2^tm1Lau^) were obtained from the University of Campinas’ breeding center. All experiments were performed in accordance with the guidelines from the Brazilian College for Animal Experimentation and were approved by the Animal Research Ethics Committee at the University of Campinas (#2016-1). The animals were kept on artificial light-dark cycles. Rodents were randomly divided into two groups: lean mice which fed on standard rodent chow (3.948 kcal.kg^−1^), and diet-induced obesity (DIO) which fed on fat-rich chow (5.358 kcal.kg^−1^) *ad libitum* for 3 months. This diet composition is described in Table S1.

### Intracerebroventricular cannulation (ICV)

**Figure 5:**
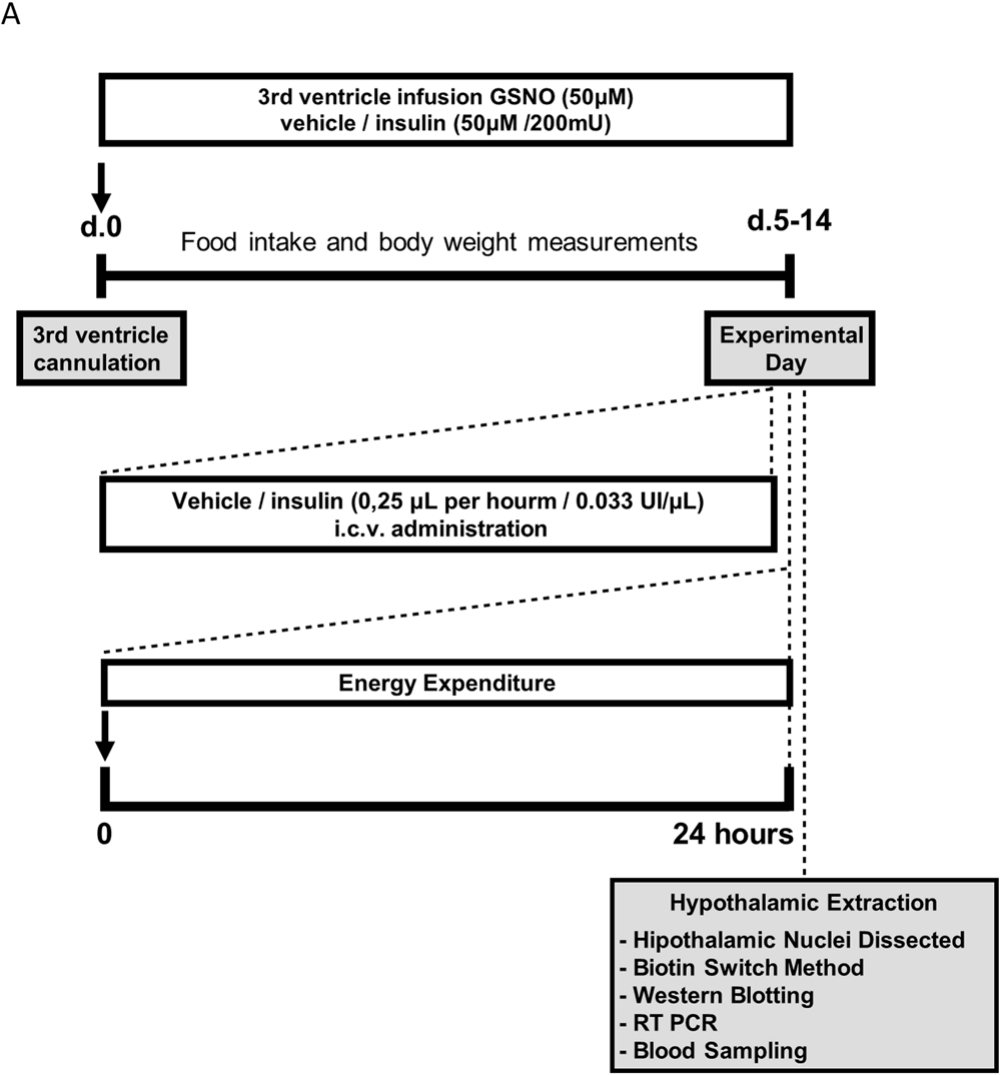
Experimental design.

Animals were submitted to stereotaxic placement of a stainless steel indwelling guide cannula into the third ventricle (stereotaxic coordinates for rats DV: −8.5 mm and AP: - 0.5 mm; mice DV: −5 mm and AP: −1.8 mm) under intraperitoneal anesthesia [mix of ketamine (10 mg) and diazepam (0.07 mg) (0.2 ml/100 g body weight)]. A mini-osmotic pump (model 1002, Alzet®, DURECT Corporation, Cupertino, CA, USA) inserted on the animals’ dorsum was connected to the brain infusion cannulae, for chronic ICV administration of treatments (Fig. 5).

### Metabolic monitoring

Oxygen consumption/carbon dioxide production was measured using LE405 Gas Analyzer and spontaneous locomotor activity was detected by a computer-controlled system (Panlab – Harvard Apparatus, Holliston, MA, USA) as previously described^60^.

### Exercise protocols

After acclimated to swimming for 2 days (10 min per day), rats performed two 3 h exercise bouts, separated by one 45 min rest period, as previously described^61^. Exercise protocols finished at 6:00 p.m. for posterior measurement of food intake and extraction of hypothalamic tissue for evaluation of protein expression, phosphorylation and S-nitrosation.

### Intracerebroventricular treatments

All intracerebroventricular infusions were performed using a mini-osmotic pump (model 2002, Alzet®, DURECT Corporation, Cupertino, CA).

### S-nitrosoglutathione (GSNO)

Solid GSNO was synthesized in advance by reacting glutathione with sodium nitrite in acidic solution, as previously described^41^. On the experiment day, solid GSNO was dissolved in PBS pH 7.4 resulting in a GSNO 50 µM solution^62^. Chronic administration of GSNO was performed by ICV infusion of an aqueous GSNO solution 50 µM at a flow rate of 0.25 µL/h for 8–14 days.

### Insulin

Animals received ICV infusion of insulin (200 mU) at 6:00 p.m. to evaluate food intake or insulin signaling and 24 hours food intake was measured. Chronic administrations of GSNO (50 µM)/insulin (0.033 UI/µL) and GSH (50 µM)/insulin (0.033 UI/µL) were performed by ICV injections of the mixed solutions using an osmotic pump for 7 days.

### iNOS antisense oligonucleotide (ASO)

Phosphorothioate-modified sense and antisense oligonucleotides (produced by Invitrogen Corp., Gaithersburg, CA, USA) were designed according to the iNOS sequence deposited at the NIH-NCBI (http://www.ncbi.nlm.nih.gov/entrez) and were composed of 5′-GCA TAC CTG AAG GTG-3′ (sense) and 5′-GCA TAC CTG AAG GTG-3′ (antisense). iNOS ASO or iNOS sense (100 mM) sense were administered at a flow rate of 0.25 µL/h for 5 days.

### L-N6-(l-iminoethyl) Lysine (L-NIL) treatment

Lean and obese rats were treated daily with pharmacological inhibitor of iNOS (L-NIL) or saline, injected chronically via ICV at a flow rate of 0.25 µL/h for 24 h.

For Western blotting and S-nitrosation analysis, after L-NIL treatment, rats were submitted to fasting for 8 h and received ICV infusion of insulin (200 mU) to determine insulin signaling.

### Serum insulin, leptin, and cytokines quantification

Blood was collected from the cava vein 15 min after the exercise protocols or 10 min after ICV treatments. Plasma was separated by centrifugation (1.100 g) for 15 min at 4 °C and stored at −80 °C until assay. Serum insulin concentration was determined using a commercially available Enzyme Linked Immunosorbent Assay (ELISA) kit (Crystal Chem Inc., Chicago, IL, USA). Leptin, triglycerides, adiponectin, TNF, IL-6, IL-1β and IL-10 were measured using ELISA following the manufacturer’s instructions.

### Quantification of GSNO decomposition in hypothalamic homogenate

The decomposition of GSNO after its incubation with hypothalamic homogenate at an initial concentration of 50 μM was measured by chemiluminescence using a nitric oxide analyzer (Sievers NOA 280i, GE Analytical Instruments, Bolder CO, USA), operating at O2 and N2 pressures of 6.0 psi and 7.0 Torr, respectively. The instrument was previously calibrated with standard GSNO solutions and NO was quantitatively released from GSNO in a reduction reaction with ascorbic acid at pH 11, as described elsewhere^63^. The amount of GSNO remaining in the hypothalamic homogenate was quantified after 20 and 60 minutes of incubation at 37 °C and compared with a control (GSNO incubated in PBS solution, pH 7.4 in the same conditions). Measurements were performed in triplicate and are expressed as the mean ± SD.

### Protein analysis by immunoblotting

The hypothalamus and hipothalamic nuclei were dissected as previously described^64^ and homogenized in a modified lysis buffer (1% Triton X-100, 100 mmol/l Tris pH 7.4, 100 mmol/l sodium pyrophosphate, 100 mmol/l sodium fluoride, 10 mmol/l EDTA, 10 mmol/l sodium vanadate, 2 mmol/l phenylmethylsulphonyl fluoride, and 0.1 mg aprotinin). Proteins were separated by SDS-PAGE and transferred to nitrocellulose membranes. The proteins of interest were detected using specific antibodies and SuperSignal^TM^ West Pico Chemiluminescent Substrate (Thermo Scientific). Band intensities were quantified by using Image Lab™ Software for PC Version 6.0 (Bio-Rad laboratories Inc.).

### Detection of S-nitrosated proteins by Biotin Switch Method

Detection of S-nitrosated proteins were performed as previously described^65^. Briefly, hypothalamic tissue was extracted and homogenized in extraction buffer (250 mM Hepes, pH 7.7, 1 mM EDTA, 0.1 mM neocuproine) adjusted to 0.5 mg/ml and then blocked with a four volumes of blocking buffer [225 mM Hepes, pH 7.7, 0.9 mM neocuproine, 2.5% SDS, and 20 mM methyl methanethiosulfonate (MMTS)]. After, adding acetone to precipitate and remove MMTS excess, ascorbic acid and biotin-HPDP were used to reduce the S-NO groups and label the proteins. Labeled proteins were precipitated with neutravidin–agarose beads and then separated by SDS-PAGE, electrophoretically transferred to nitrocellulose membranes and the proteins of interest were detected using specific antibodies and SuperSignal^TM^ West Pico Chemiluminescent Substrate (Thermo Scientific™). Band intensities were quantified by using Image Lab™ Software for PC Version 6.0 (Bio-Rad laboratories, Inc.).

### mRNA isolation and Real-Time PCR

Hypothalamic tissue was homogenized in Trizol reagent for RNA extraction (Life Technologies, Gaithersburg, MD, USA). Reverse RNA transcription was performed using Maxima First Strand cDNA Synthesis Kit with dsDNase for quantitative PCR (Thermo Scientific™). Real-time PCR was performed using TaqMan RT-PCR Master Mix (Applied Biosystems), as follows Gene Expression probes: proopiomelanocortin (POMC): Rn00595020_m1, neuropeptide-Y (NPY): Rn00561681_m1, agouti-related peptide (AgRP): Rn01431703_g1, cocaine-and amphetamine-regulated transcript (CART): Rn01645174_m1 and glyceraldehyde-3-phosphate dehydrogenase (GAPDH) (#4352338E; Applied Biosystems) was used as an endogenous control.

### Statistical Methods

The differences between the means were analyzed using Prism (Graphpad) and STATA (StataCorp) and assessed by the Student’s t-test, multiple measures ANOVA or by one-way ANOVA, followed by post hoc analysis of significance (Bonferroni posthoc test) as indicated. The level of significance was set at *p* < 0.05.

## Electronic supplementary material

Supplementary Figures and Tables
