# Supplementary figures and images for: iNOS promotes hypothalamic insulin resistance associated with deregulation of energy balance and obesity in rodents
Source: Sci Rep. 2017 Aug 23;7:9265. doi: 10.1038/s41598-017-08920-z (PMC5569114; doi:10.1038/s41598-017-08920-z)

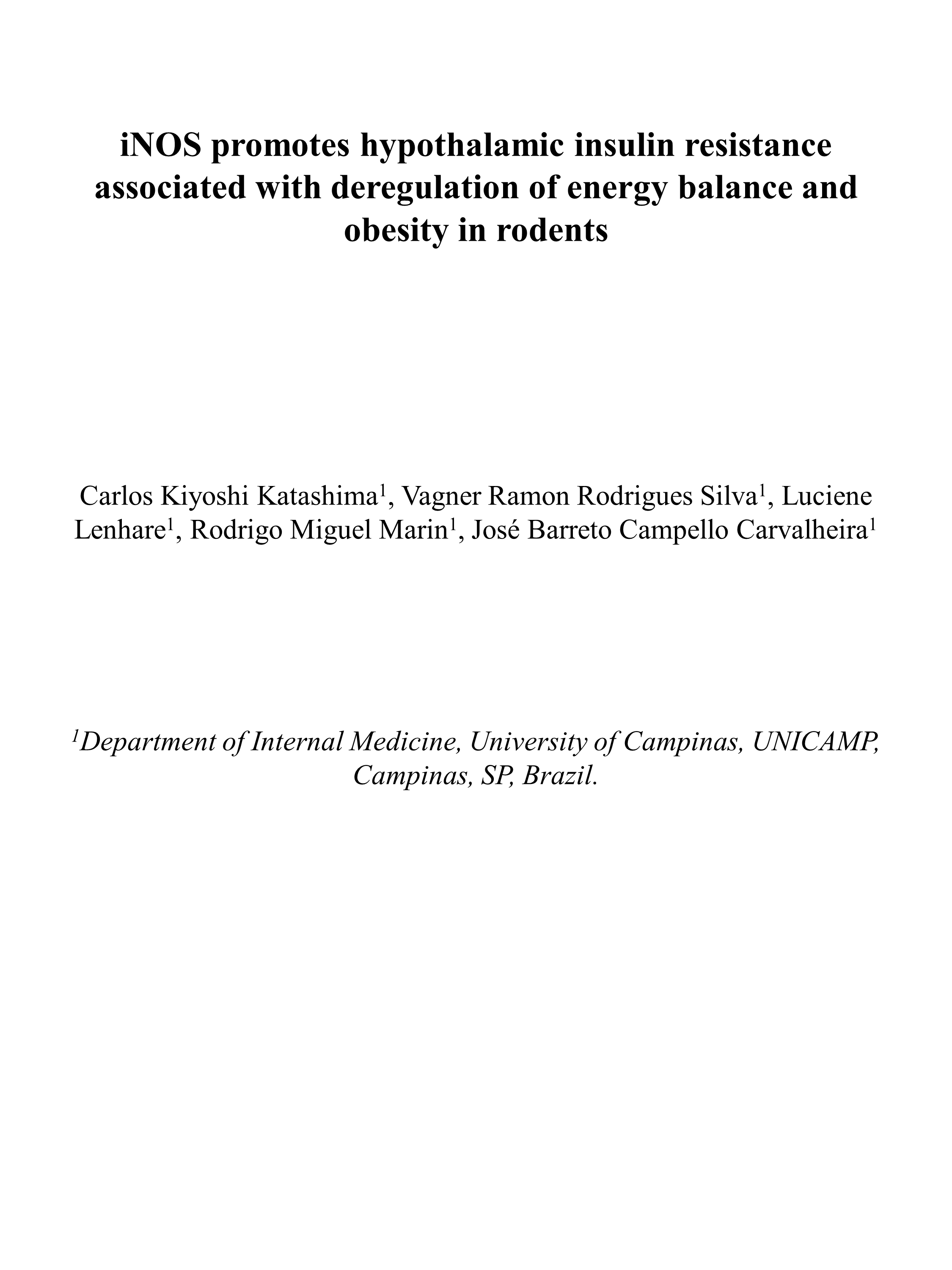

Supplement: Supplementary file 1 — Supplementary Figures and Tables [file 41598_2017_8920_MOESM1_ESM.tif]
